# The Association Between Parents’ and Adult Children’s Homeownership: A Comparative Analysis

**DOI:** 10.1007/s10680-015-9351-3

**Published:** 2015-11-26

**Authors:** Clara H. Mulder, Caroline Dewilde, Mark van Duijn, Annika Smits

**Affiliations:** Population Research Centre, Faculty of Spatial Sciences, University of Groningen, P.O. Box 800, 9700 AV Groningen, The Netherlands; Department of Sociology, Tilburg University, Tilburg, The Netherlands; Department of Spatial Economics, VU University, Amsterdam, The Netherlands; Tinbergen Institute, Amsterdam, The Netherlands; University of Amsterdam, Amsterdam, The Netherlands; Department of Economic Geography, University of Groningen, Groningen, The Netherlands

**Keywords:** Intergenerational transmission, Homeownership, Europe, Discrete-time event history analysis

## Abstract

We investigate the extent to which the intergenerational transmission of homeownership varies across European countries. Our main hypotheses are that the impact of parental homeownership on the likelihood and timing of an adult child’s entry into homeownership is less strong in contexts where homeownership is more accessible (in terms of affordability and access to mortgage credit), where renting is a feasible alternative to owning, and where the family matters less for the provision of welfare and housing. We perform discrete-time event history analyses of the transition to first-time homeownership using retrospective SHARELIFE-data from 10 European countries. Our respondents were born between 1908 and 1963, while observed entries to first-time homeownership occur between 1965 and 2009. We introduce fixed effects for countries and macro-level indicators for country-period combinations, interacted with parental homeownership. We find that the intergenerational transmission of homeownership is stronger in contexts where house prices are higher (and homeownership is less affordable), and less strong in more affluent contexts and in contexts where homeownership has increased more. The remaining differences in intergenerational transmission cannot be attributed to differences in welfare regimes or between dual and unitary rental markets.

## Introduction

Living in an owner-occupied dwelling has an indisputable influence on people’s lives. Not only does homeownership provide for economic security and wealth, it also represents social status (e.g., Dietz and Haurin 2003) and has great emotional value (Saunders 1990). Homeownership is also an area in which social inequality is reproduced: Children of homeowners are more likely to become homeowners than children of parents who live in rented housing (e.g., Aratani 2011; Boehm and Schlottman 1999; Henretta 1984, 1987; Mulder and Smits 1999; Mulder and Wagner 1998; Smits and Mulder 2008). Intergenerational continuities in homeownership form part of a complex system of intergenerational transmission of wealth and other resources, possibly resulting in widening inequalities between households’ standards of living and wealth levels. Such cumulative inequalities, over and above inequalities arising from the labor market, arise to the extent that homeownership is associated with additional economic benefits compared with renting, enhancing opportunities for wealth accumulation (e.g., Kurz and Blossfeld 2004a, b).

The occurrence and timing of the transition to first-time homeownership differ across Europe (Kolb et al. 2011; Scanlon and Whitehead 2004). Whether and when people make this transition highly depends on the accessibility of homeownership—there has to be a sufficient supply of affordable owner-occupied dwellings—and on the presence of feasible alternatives. An important individual characteristic influencing the likelihood of becoming a homeowner is whether one’s parents are homeowners. Differences in the importance of intergenerational transfers are likely to account partly for differences in homeownership transitions between countries.

Most studies addressing the access to homeownership in different countries or welfare regimes focus on housing provision systems, mortgage markets or other macro-level factors (Hoekstra 2005; Kemeny 1995; Mulder and Billari 2010). With the exception of the literature on southern Europe—where homeownership is often outright, achieved by self-provisioning through the pooling of family resources and imbedded within the context of the exchange of care for the intergenerational transmission of housing wealth (e.g., Allen et al. 2004; Poggio 2008)—the role of the family with regard to cross-national differences in access to homeownership has remained somewhat neglected. From the available evidence, however, it can be expected that family assistance matters in different ways across different time periods and institutional contexts. Using SHARELIFE-data, Angelini et al. (2013) found sizeable differences in the means of acquiring homeownership across European countries. Family assistance was more common in Italy, Greece, Switzerland, Germany, Austria, Poland and the Czech Republic than in Sweden, Denmark, the Netherlands, Belgium, France and Spain.

The intergenerational transmission of homeownership—that is, the impact of parental homeownership on their adult children’s transitions to homeownership—has mainly been studied for single countries, including the USA (Henretta 1984, 1987), the Netherlands (Helderman and Mulder 2007; Mulder 2004; Mulder and Smits 1999; Smits and Mulder 2008), West Germany (Kurz 2004), Denmark (Leth-Sørensen 2004), Israel (Lewin-Epstein et al. 2004) and Italy (Poggio 2008). Mulder and Wagner (1998) found a stronger impact of parental homeownership for West Germany than the Netherlands. An indication of differences between contexts can also be derived from the effects of parental homeownership in comparative case studies (e.g., Kurz and Blossfeld 2004a). These effects, however, were estimated using datasets that differed in design and in the availability of control variables.

There is also evidence of an increasing importance of parental homeownership to adult children’s transitions to homeownership for more recent birth cohorts (Enström Öst 2012; Kurz and Blossfeld 2004b; Forrest 2013). For the cohorts under study in this paper (entering homeownership between 1965 and 2009), these trends have been linked to worsening macro-economic conditions since the mid-1970s influencing the labor market position and timing of family formation of young people and to housing market-related determinants, in particular decreasing affordability, influencing the access to homeownership. We contribute to the existing literature by focusing on and testing the impact of housing market-related determinants (affordability, access to mortgages) in a more formal way with a better research design.

More rigorous cross-country comparisons of the effect of parental homeownership on transitions to first-time homeownership can be achieved using internationally comparable, harmonized data on multiple countries that contain information about housing histories, parental homeownership and longitudinal data on other important factors that might explain the transition to homeownership. Until recently, no such data were available. The third wave of the Survey of Health, Ageing and Retirement in Europe (SHARE), collected in 2008/2009 for individuals aged 50 and over and their partners and known as SHARELIFE, contains the necessary information in the form of retrospective life histories of individual respondents in 13 European countries.

The aim of this paper is to investigate differences in the occurrence and timing of the transition to first-time homeownership between countries and across historical time. In particular, we analyze the impact of parental homeownership on this transition and how this impact differs across countries. We conduct discrete-time event history analyses (logistic regression of person-year data) of the transition to first-time homeownership for ten countries: Sweden, Denmark, the Netherlands, Germany, Switzerland, France, Belgium, Italy, Spain and Greece. Besides the variables measuring individual and family characteristics, our models contain country fixed effects, macro-level indicators referring to country-period combinations and interactions between these combinations and our indicator of parental homeownership.

## The Intergenerational Transmission of Homeownership

In the literature, four major mechanisms have been identified through which the intergenerational transmission of homeownership takes place: (1) parental housing assistance, (2) tenure transmission as a side effect of socioeconomic status transmission, (3) geographical proximity between the two generations and (4) socialization toward homeownership (Helderman and Mulder 2007; Lersch and Luijkx 2015).^1^

Parental assistance toward homeownership usually takes the form of gifts, loans or mortgage guarantees (Helderman and Mulder 2007; Mulder 2007), although it may also include non-financial forms such as help with self-building. Homeowning parents are more likely to help their adult children financially than parents who rent their accommodation (Grundy 2005; Mulder and Smits 1999, 2013). They often have lower housing costs because they are outright owners or approaching outright ownership (Haffner 2008). This gives them the opportunity to accumulate savings and to spend part of their savings on assisting their children. They could also release equity from their property to assist a child.

Parents with a high socioeconomic status have more financial resources to invest in their children than those with a low socioeconomic status. They are also more likely to own their houses. Consequently, children of high-status parents, and therefore homeowner parents, have better opportunities for acquiring homeownership than children of low-status parents. In a study for the Netherlands, however, the effect of parental homeownership hardly diminished after taking into account parental socioeconomic status (Helderman and Mulder 2007).

Parents and children often do not live far apart (Glaser and Tomassini 2000; Malmberg and Pettersson 2007; Michielin and Mulder 2007)—either because they wish to remain close or because long-distance moves are rare in general—and hence, it is likely that they operate in the same housing market. When owner-occupied homes prevail in a certain area, and both parents and children live in that particular area, it is likely that both are homeowners. The association between parents’ and children’s housing tenures is therefore partly explained by the tenure structure of the local housing market (Helderman and Mulder 2007). Homeownership is more common in rural areas and less common in large cities (Mulder and Wagner 1998).

Expectations about future living conditions and preferences for certain life styles are developed early in the life course. It is therefore likely that growing up in an owner-occupied home—which is usually larger and of higher quality than a rented home—might lead to a preference for homeownership later in life through socialization (Henretta 1984; Semyonov and Lewin-Epstein 2001). Lersch and Luijkx (2015) tested the socialization hypothesis by taking into account the length of time spent in parental homeownership while controlling for several indicators of parental wealth. They found that the likelihood of entering homeownership, and of being a homeowner in later life, was substantially increased by each additional year spent in homeownership during childhood. Socialization might also take the form of an active process. As Mulder and Smits (2013) argued, homeowner parents might be particularly keen on assisting their adult children in becoming homeowners rather than just giving them money and therefore dedicate gifts toward homeownership. Their empirical findings, however, did not provide support for this idea.

The main question of this paper regards contextual factors across time and space causing variation in the intergenerational transmission of homeownership. The four different underlying mechanisms of this transmission may reinforce or counteract each other and may be more or less important across European countries and different time periods. To reduce complexity, our contextual-level theoretical focus mainly lies with the differences that influence the need for or the level of parental assistance. We hence assume that the impact of parental assistance is proxied by the impact of parental homeownership. Previous research (e.g., Kurz 2004) has also been based on this assumption.

## Hypotheses: Contextual Variation in the Intergenerational Transmission of Homeownership

Given the various mechanisms through which homeownership is transmitted between generations, our first hypothesis is that parental homeownership has a positive impact on the transition to homeownership in all countries under study. The size of this impact is likely to differ across countries and through historical time. Our hypotheses about how these differences take shape are based on ideas on (1) access to homeownership—and thus the extent to which parental support is necessary or helpful to achieve homeownership, (2) the extent to which renting forms a feasible alternative to owning and (3) welfare regimes and family systems—and thus the extent to which institutional and cultural factors are in line with a strong versus weaker role of the parents in the younger generation’s homeownership. For a stylized overview of country classifications, please refer to Table 1.

**Table 1:** Country classification of rental markets (unitary vs. dual) and welfare regimes

	Rental market		Welfare regime		
	Unitary	Dual	Social-democratic	Conservative	Southern European
Netherlands	X		X		
Sweden	X		X		
Denmark	X		X		
Germany	X			X	
Switzerland	X			X	
France		X		X	
Belgium		X		X	
Italy		X			X
Spain		X			X
Greece		X			X

### Access to Homeownership

Access to homeownership is determined by the opportunities to become a homeowner and the constraints making the transition to homeownership difficult; both opportunities and constraints differ across countries and through time. Our general hypothesis is that difficult access to homeownership leads to greater dependence on parents and, therefore, a stronger association between parental homeownership and adult children’s likelihood of becoming homeowners. House prices are obviously an important determinant of access to homeownership, particularly in relation to income or affluence—the combination of both defines the concept of ‘housing affordability.’ In periods and countries when and where house prices are high, relative to income, we expect a stronger impact of parental homeownership on transitions to homeownership than when homeownership is more affordable. Affluence itself may also be important. Access to homeownership may be easier in affluent societies. The historical increase in homeownership rates is often associated with the rise of economic affluence following World War II (Harloe 1995; Saunders 1990). It is, however, not clear what association we should expect between the level of affluence of societies and the intergenerational transmission of homeownership. In affluent societies, children might be less in need of parental help. But parents may also be in a better position to accumulate savings, which would allow them to pass a larger part of their wealth onto their children.

Access to mortgage credit also affects access to homeownership and differs considerably across Europe (Mulder and Billari 2010). In periods and countries with poorly developed mortgage markets, where loan-to-value ratios are low and thus down-payments high, access to homeownership is restricted to those who have savings and/or those who receive financial help from family in the shape of gifts, loans or bequests. It should be noted that in more recent times, mortgage deregulation in a number of countries has resulted in a higher availability of credit, driving up house prices and house price volatility (e.g., Andrews and Caldera Sánchez 2011), so that children might still revert to parents for down-payments, co-signing loans or help with mortgage repayments. This is, however, more of a problem for the younger generations entering homeownership since roughly the late 1990s and less of a problem for the cohorts under study in this paper. We therefore hypothesize that in countries where mortgage credit is more readily available, the association between parents’ and children’s homeownership will be smaller.

### Renting as an Alternative to Owning

In contexts where renting is viewed as an alternative to owning, there is less need for parental assistance to support adult children’s homeownership than in contexts where homeownership is viewed as the ‘better’ tenure (e.g., in terms of economic benefits, or as a more suitable type of housing for raising children). A useful framework for distinguishing between contexts according to the prevailing—often ideological—views on renting versus owning was offered by Kemeny (1981, 1995). He pointed out that housing tenure structures are the result of political choices influencing the relative costs and benefits of owning versus renting. He made a distinction between unitary and dual rental markets, which may have different consequences for young adults’ housing pathways.

In ‘unitary’ rental markets such as the Netherlands, Sweden, Denmark, Germany and Switzerland, the costs and benefits of renting versus owning are more similar, and housing subsidies for all stakeholders blur housing-related segregation between social classes. Private landlords were allowed to enter a state-managed market ‘in which rent-setting and tenancy rights were overseen and mediated by the wider state in exchange for access to state subsidy’ (Lowe 2011: 139). As a consequence, the competition and often less clear-cut distinction between larger, but more strongly regulated, public and private rental sectors result in good-quality housing across tenures for different income groups (Kemeny 1981). Thus, in these countries, suitable homes for middle-income groups can also be found in the rental sector, and households can more easily base their tenure choices on their life-course stage. The need for parental assistance is therefore likely less strong in these countries than compared with countries that can be characterized as having a dual rental market.

In countries with a dual rental market, renting is more of a ‘residual’ tenure: In our study, this concerns Belgium, Italy, Spain, Greece and, to a lesser extent, France. Housing policies in these countries have, for various reasons, mainly focused on solving housing needs by means of private homeownership (e.g., Lowe 2011; Goossens 1983). A myriad of subsidies and regulations has made homeownership accessible to large parts of the population. Dual rental market countries are characterized by a smaller rental sector in general, along with a strong divide between an unregulated private rental sector and a (often smaller) social rental sector, with the latter being mainly targeted at low-income groups (Kemeny 1995). In general, the smaller the non-profit sector, the more deprived its tenants, the more allocation is based on criteria of need and deservingness, and the higher the level of stigmatization (Lowe 2011). Private renting in these countries is furthermore more strongly associated with a lower socioeconomic position and a less favorable price/quality ratio (Winters and Elsinga 2011) and is therefore also a less attractive housing option.

Within the group of countries with a dual rental market, the southern European countries have a different history compared to, for example, Belgium or the UK (which is not in our sample). According to Allen et al. (2004), the strong growth of homeownership in southern Europe in the post-war period can be linked to the existence of strict rent controls which were sustained over a long periods of time. Rent regulation negatively affected profitability, and hence investment, in private rental housing—leading to a strong decline of the tenure and its housing quality over time. In Spain and Italy, significant amounts of public housing units were furthermore sold to sitting tenants. Hence, the driving force of increasing homeownership rates in southern Europe was not so much the extent of active support for this tenure, but rather the conscious lack of government support of the other tenures, leaving no other option but homeownership for large parts of the population. Gaps in housing provision were subsequently solved within the extended family. ‘Informal’ cheap routes to self-provisioned homeownership were sustained by weak or weakly enforced land use and building standard regulations until the 1980s, within a context of economic restructuring and internal migration (Poggio 2013; Cabré Pla and Módenes Cabrerizo 2004). This rapid expansion of homeownership in southern Europe transformed these societies from renting nations to owning nations, be it of a specific nature.^2^ In recent decades, stricter regulation has resulted in a shift toward more a market-based production of homeownership (for Italy, see Poggio 2013). Access to homeownership became less affordable; combined with difficulties to secure a good labor market position leading to delayed family formation, this has resulted in later ages of nest-leaving (and consequently first-time entry into homeownership) compared to other European countries.

Based on the arguments outlined above, we expect that in countries with a dual rental market, parental homeownership has a stronger impact on tenure outcomes than in countries with a unitary rental market. Roughly since the 1990s, there has been a trend toward a higher reliance on market mechanisms in unitary rental markets. Although pressures toward a more market-orientated regime play out quite differently in different countries (e.g., Ruonavaara 2011), the distinction between unitary and dual rental markets remains important, in particular for the time period under study in this paper.

### Welfare Regimes and Family Systems

Although Esping-Andersen (1990, 1999) did not address housing in his major contributions to welfare regime theory, his work contributed to the understanding of country differences in the intergenerational transmission of homeownership. He pointed at the different relationships between state, market and family, as the main providers of welfare in social-democratic, liberal and conservative welfare regimes. A fourth regime can be added: the southern European regime (Ferrera 1996). In this regime, the family plays an important role in the provision of welfare, often as a consequence of ineffective state policies (Castles and Ferrera 1996). The particularly strong role of the family in southern Europe is also stressed in Reher’s (1998) work on family systems. Of the countries in our data, the Netherlands, Sweden and Denmark represent the social-democratic regime; Germany, Switzerland, France and Belgium represent the conservative regime; and Italy, Spain and Greece represent the southern European regime (which coincides with a more strongly family-oriented system). It should be noted that the Netherlands has characteristics of both the conservative and the social-democratic regimes and could be classified as either. With regard to housing provision, however, the role of the state was quite strong in the Netherlands in the period under study, and for this reason, we think that classification as a social-democratic welfare regime is justified.

Differences in the extent to which homeownership is transmitted from parents to children are likely to be associated with differences in welfare regimes. There are four reasons to expect a stronger effect of parents’ on adult children’s homeownership in conservative and particularly southern European welfare states. First, family help in general, and parental housing assistance in particular, is likely to matter more in countries with a conservative and particularly a southern European welfare regime. In these countries, the family plays an important role in the provision of welfare, including housing. In southern European countries, the family ‘tends to operate as a clearinghouse for the pooling of social and material resources and for their redistribution among its members according to need’ (Castles and Ferrera 1996, p. 181). For making the transition to first-time homeownership, southern European households are much more dependent on personal savings and on family wealth than households in other parts of Europe (Poggio 2008). Besides financial assistance, other forms of parental inter vivos transfers are more common as well (Poggio 2008): plots of land, existing family dwellings and transfers in the form of labor while assisting with the self-building of a home. Assistance with self-building is quite common in southern European countries and to a lesser extent also in conservative welfare regimes, particularly in rural areas (e.g., Kurz 2004).

Second, the role of socioeconomic status transmission, accounting for part of the homeownership transmission, might differ between countries. Socioeconomic status transmission can be expected to be smaller in countries with more state support, where social policy is explicitly based on principles of equality and redistribution, counteracting the formation of class-based monopolies and social closure (e.g., Esping-Andersen 1990; Silver 1994). In conservative countries, social policies were historically developed to maintain, rather than mitigate differences between classes and status groups. In liberal countries, parental resources are equally likely to matter more, given the prominence that is given to market provision.

Third, the geographical distance between parents and children is, in general, much smaller in southern European countries than in other European countries (Hank 2007). This smaller distance could be related to the strong family systems in these countries: The great importance attached to the family, combined with a higher reliance on family help as a form of welfare provision, could lead to reluctance to move away from family members. When parents and children live nearby, they operate in the same housing market, which can be dominated by owner-occupied housing. The distance between parents and children does not only reflect differences in housing market characteristics. Large intergenerational distances have also been found to influence the gift-giving behavior of parents: More money is transferred to children who live close (Tomassini et al. 2003). We note that the causal direction of the relation between parental housing assistance and intergenerational proximity is debated (Poggio 2008). Nonetheless, this association implies that the likelihood of the intergenerational transmission of homeownership decreases with distance; partly as a result of housing market similarities and partly as a result of parental gift-giving. These distances are smaller in the southern European welfare regime.

Fourth, as Albertini et al. (2007) argue, there seem to be different patterns in intergenerational transfers of time and money that largely follow welfare regimes. Transfers from parents to children seem to be less frequent but involve higher amounts in the southern European than the Nordic countries, with the Continental European countries in between.

## Other Contextual Differences and Individual Factors Influencing the Transition to Homeownership

Besides those we used in our hypotheses, numerous other contextual differences could influence the intergenerational transmission of homeownership or the likelihood of a transition to first-time homeownership. First of all, we account for changes in levels of homeownership through time. In countries where homeownership has increased strongly between generations, the association between parents’ and their children’s homeownership simply cannot be very strong—people cannot pass on what they do not have. In particular for our older respondents, a larger part of their parents did not own their homes at the time of the child’s house purchase. After all, at the turn of the 20th century, most people lived in private-rented dwellings, of low quality and at high cost. Following pressures caused by industrialization and urbanization (low incomes, housing shortages, slum dwellings), housing provision increasingly moved into the realm of public policy, resulting in a gradual shrinkage of private renting (e.g., Lowe 2011). In some countries, homeownership was promoted early on, and in others housing policy goals were realized through rent regulation and social housing.

While social housing peaked in the post-war decades and has declined in many countries since the 1970s, homeownership rates more or less steadily increased across most of Europe during the course of the 20th century. This can be seen from the available time series data (mostly from 1947; Martens 1985, for the Netherlands, Denmark, France and West Germany; chapters for separate countries in Kurz and Blossfeld 2004a, for Italy and the Flemish part of Belgium). West Germany is an exception, with a decrease between 1947 and the mid-1950s and an increase afterwards. Strong increases in homeownership levels following World War II are found for the southern European countries in particular (see also Allen et al. 2004).

We also account for the level of homeownership itself, as the likelihood of a transition to homeownership should logically vary with the availability of the tenure. No doubt, other contextual differences that we cannot account for are also important. Examples of these may be the extent to which homeownership or renting is promoted by the government through tax relief or subsidies (although this is partly captured by the difference between unitary and dual rental markets) and the fiscal treatment of gifts from parents to children.

Several individual- and household-level factors are known to influence the likelihood of a transition to homeownership (Henretta 1984, 1987; Kurz 2004; Leth-Sørensen 2004; Lewin-Epstein et al. 2004; Mulder 2004; Mulder and Smits 1999; Mulder and Wagner 1998; Poggio 2008; Smits and Mulder 2008) and are taken into account. Homeownership can only be achieved with sufficient financial resources. The most important source of financial resources is paid work. Whether someone has paid work is important, but also the level of the job, income potential (indicated by level of education) and time spent in the labor market (an indicator of work experience and therefore income, and also of available savings). Age should be accounted for too, as for example because older people who have not become homeowners yet might belong to a specific category that is not interested in homeownership or cannot afford it. It is also important to account for partnership status and whether someone has children. Cohabiting couples, never-married singles, divorced and widowed singles are known to become homeowners less frequently than married couples. The presence of children makes owning a home more attractive and therefore possibly more likely, but the cost of children may also compete with the cost of owning a place. Finally, as Blaauboer (2010) has shown, the impact of family background, resources and household context on homeownership differs between men and women.

## Data and Method

### Data

The data were taken from the third wave of the SHARE: SHARELIFE. The SHARELIFE-data were collected in 2008/2009 among a random sample of individuals aged 50 and over and their partners, in 13 European countries: Austria, Belgium, Czech Republic, Denmark, France, Germany, Greece, Italy, the Netherlands, Poland, Spain, Sweden and Switzerland. People living in institutions were not included in the sample. The data contain retrospective information on the life histories of 26,836 respondents.

As in previous studies (e.g., Mulder and Smits 1999; Mulder and Wagner 1998; Smits and Mulder 2008), the transition to homeownership is defined as the first time someone moves into an owner-occupied home as a member of an independent household, that is, without their parents. This definition is unproblematic in countries where the vast majority of transitions to ownership are accompanied by a residential relocation. It is also the only definition we can use, because the SHARELIFE-questionnaire is not suited to measure transitions to homeownership that do not coincide with a move. Transitions to homeownership of those who remain in their parental home and become owners after their parents die or after ownership is otherwise transferred from parent to child can unfortunately not be observed. This could be problematic in countries where many people remain in the parental home until older ages. In most SHARELIFE-countries, only a small proportion of respondents still lived in the home where they were born when they were interviewed, but this proportion was larger in Austria (12.6 %), Poland (15.6 %) and the Czech Republic (15.8 %). Transitions to ownership through a tenure change of a rental home already inhabited by the respondent (e.g., Right-to-Buy policies) are equally difficult to trace. This is problematic in former socialist countries, where many homes in the public rental sector were privatized around the transformation to market economies and became owner-occupied (Kok 1999)—another reason for omitting Poland and the Czech Republic.

Our target population consisted of all respondents who were not recorded as owning a home when they were 18 years old (only very few were). Fortunately, there were not too many cases with missing values on the dependent and independent variables, and these were deleted (4.6 % of the person-years). Our respondents were born between 1908 and 1963, while observed transitions to first-time homeownership occurred between 1930 and 2009. The period of observation for the analyses including macro-level variables was, however, shorter, starting from 1965. Our analyses were therefore performed on subsets of person-years starting from 1965 (see the rows ‘Observed N person years’ and ‘Observed calendar years’ in the results tables).^3^ Our actual analytical sample hence consisted of 18,043 respondents (67.2 % of the original dataset). Negligible differences in results were found when comparing the coefficients of the simplest specifications—without macro-level variables—of our model between the original dataset and our sample (results available upon request).

### Variables

To establish whether and when the transition to first-time homeownership took place, we used information on the complete housing history of the respondent. This information was gathered retrospectively. For up to 29 former and current homes, it was recorded in which year the respondent moved in and whether this was an owner-occupied home or not. Respondents could indicate whether they were the owner, member of a cooperative and tenant of the home, whether they lived there rent-free, or otherwise. We only considered the first option (being an owner) as an indicator of homeownership, not being a member of a cooperative. The year in which the respondent moved into the first owner-occupied home was used to measure the timing of the transition. Respondents were removed from the observation window after the transition to homeownership. In case the respondent did not make the transition, the observation was censored at the time of interview. The final person-year file consisted of 381,676 person-years (men and women).

To measure the extent to which homeownership was transmitted from parents to their children, we used an indicator of whether parents were homeowners when the respondent was aged ten, using the same indicator for the parents’ as for the respondent’s homeownership.

**Fig. 1:**
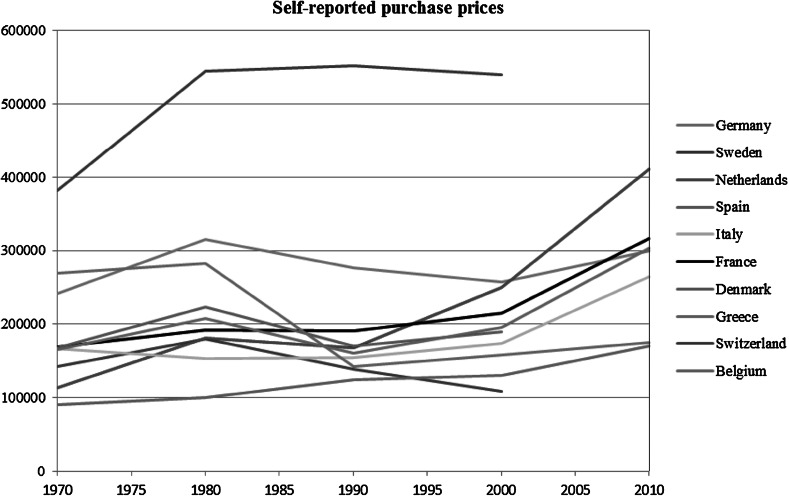
Average self-reported purchase prices in current US dollars, estimated from the SHARELIFE-data. *Note*: Three data points are missing which are Denmark, Sweden and Switzerland for the period after 2000 due to the low number of self-reported purchase prices for this period in the SHARELIFE-data

Our hypotheses on differences between unitary and dual rental markets and among welfare regimes were tested using country dummies. We introduced four macro-indicators for period-country combinations, to indicate housing affordability, affluence, access to mortgages and changes in the level of homeownership. Data on housing affordability are available from the OECD in the form of price-to-income ratios (Girouard et al. 2006). Unfortunately, however, these data are only available as indices set at 100 in 2010 for each country. As a consequence, they are well suited for within-country comparisons through time but not for comparisons between countries. We have therefore created our own housing affordability proxy, using house prices corrected for affluence. House prices were derived from the SHARELIFE-data, using information about the purchase prices of dwellings over the life courses of its respondents. Using per capita GDP-data from the United Nations Statistics Division, we calculated a price-to-GDP ratio for each country and decade from 1970 to 2010 (see Fig. 1), which we then used for adjacent years (for example, the figure for 1970 was used for 1965–1975). It is likely that this proxy contains some measurement error caused by the sample that might not correctly reflect average house prices over a period of 10 years. As a sensitivity analysis, we also used the OECD price-to-income ratios, because these are more precise even though differences between countries are not reflected. The results pointed in the same direction as those we present. The UN data for per capita GDP were also used in their own right, to measure affluence. Information about maximum loan-to-value ratios (in percentages) was taken from SHARE’s contextual database (cross-checked against OECD-data and Schwartz and Seabrooke 2008). Information about homeownership rates was taken from various sources, which were also cross-checked (Balchin 1996; Dol and Haffner 2010; European Mortgage Federation, OECD; Atterhög 2005; UN Human Settlements Database). For each country, the data consist of the percentage of homeownership starting from 1960 to 2008.^4^ These data were also used to calculate the growth rate of homeownership, measured as the percentage growth of the homeownership rate in the year of observation compared to the homeownership rate at age 10. Table 2 shows the minimum and maximum values for each macro-level indicator by country. More detailed information about the macro-level indicators can be obtained from the authors.

**Table 2:** Descriptive statistics of macro-indicators by country

	Price-to-GDP ratio	Per capita GDP	Maximum loan-to-value	Homeownership rate	Homeownership growth	Price-to-income ratio^a^
	Min.–max.	Min.–max.	Min.–max.	Min.–max.	Min.–max.	Min.–max.
Netherlands	4.7–8.3	2728–46,774	75–115	29–58	0–100	43.8–105.5
Sweden	2.8–5.9	4490–49,355	75–95	36–58	0–61	54.5–100.9
Denmark	3.7–9.3	3365–56,412	70–80	43–59	0–37	55.5–128.7
Germany	7.1–17.7	2634–39,804	65–80	29–46	0–59	99.6–186.8
Switzerland	8.8–18.8	3721–70,124	65–80	28–43	−17–51	86.8–165.1
France	5.2–11.2	2824–39,362	66–100	41–57	0–39	58.5–106.1
Belgium	4.7–11.1	2730–43,102	65–100	50–68	0–36	47.6–103.0
Italy	4.4–14.6	2049–33,968	50–80	45–73	0–62	63.5–112.3
Spain	5.3–14.3	1178–29,987	58–80	52–85	0–63	43.3–115.0
Greece	6.2–34.1	1424–26,483	50–80	70–80	−13–10	66.7–107.0
N period-country combinations	47	47	47	60	60	353

^a^Price-to-income ratio has only been used in sensitivity analysis

The occupational status of the main breadwinner parent at respondent’s age ten (the only time for which it was available in the data) was used as a measure of one of the mechanisms through which the intergenerational transmission of homeownership may take place: as a side effect of socioeconomic status transmission. In the questionnaire, the following answering categories were used: (1) legislator, senior official or manager; (2) professional; (3) technician or associate professional; (4) clerk; (5) service, shop or market sales worker; (6) skilled agricultural or fishery worker; (7) craft or related trades worker; (8) plant/machine operator or assembler; (9) elementary occupation; and (10) armed forces. These were grouped into four new categories indicating the type of job: elementary (category 9), less skilled job (categories 4 through 8), technician or associate professional (category 3), and professional (categories 1 and 2). A separate category was used to indicate that respondents reported that there was no main breadwinner parent, that they did not know what their parent’s occupation was, when the answer was missing, or when the breadwinner was in the armed forces.

Degree of urbanization at respondent’s age 10, in three categories, was used as an indicator of having grown up in a particular type of housing market with likely more ownership (rural areas and villages) or less ownership (smaller towns; large cities).

Various time-varying indicators of the respondent’s own resources were included. The first was the type of the respondent’s current or, if the respondent did not work, the last job. This was measured in the same way as the breadwinner parent’s job level, but with separate categories for the armed forces and for when the respondent had never had a job. Employment status was categorized in employed, non-employed and in education. Years in the labor market was measured as the number of years the respondent had spent in paid work. Level of education was measured using the number of years the respondent spent in full-time education from age 12 up to the year of observation.

We included the respondent’s age as a control variable. The partnership history of the respondent was used to determine whether the respondent was in a partnership at each point in time. Using the years of entering and ending a partnership, we created a series of dummy variables indicating whether the respondent was married, cohabiting unmarried, never partnered, widowed or divorced in each successive year of the person-period file. Whether the respondent had children was also introduced as a time-varying variable, measured in three categories: no children, one or two children and three or more children. Descriptive statistics of the individual-level variables, including those for parents, are reported in Table 3.

**Table 3:** Descriptive statistics (*N* = 18,043; last observed person-year)

	Mean	SD	Minimum	Maximum
Transition to first-time homeownership	0.71		0	1
Parents homeowner at age 10	0.58		0	1
Parent’s job at age 10				
Elementary	0.19		0	1
Less skilled job	0.64		0	1
Technician/ass. professional	0.05		0	1
Professional	0.09		0	1
Unknown/armed forces/no breadwinner	0.04		0	1
Degree of urbanization				
Rural/village	0.40		0	1
Moderately urban	0.44		0	1
Large city	0.16		0	1
Current or last job				
Elementary	0.10		0	1
Less skilled job	0.36		0	1
Technician/ass. professional	0.07		0	1
Professional	0.11		0	1
Unknown/armed forces	0.01		0	1
Never paid work	0.35		0	1
Employment status				
Employed	0.62		0	1
Non-employed	0.37		0	1
In education	0.02		0	1
Years in labor market	17.04	14.40	0	73
Education (in years)	5.27	4.06	0	16
Age	43.57	18.21	18	100
Year of birth	1942.01	9.90	1908	1963
Marital status				
Married	0.76		0	1
Never partnered	0.06		0	1
Cohabiting unmarried	0.07		0	1
Widowed/divorced	0.11		0	1
No children	0.32		0	1
1–2 children	0.50		0	1
3 or more children	0.18		0	1

*Source*: Third wave of the Survey of Health, Ageing and Retirement in Europe (SHARE): SHARELIFE

### Method

We performed a series of discrete-time hazard analyses of the transition to first-time homeownership, by means of Logistic regression of person-years, for men and women separately.^5^ In the first model, the independent variables were parental homeownership, fixed effects for countries (nine dummies) and an interaction between parental homeownership and the country dummies to assess differences between housing markets and welfare regimes in the intergenerational transmission of homeownership. In the second model, we added independent variables related to the individual and his or her parents. Next, we estimated models in which the macro-level indicators (estimated for period-country combinations) were added one at a time, as a main effect and interacted with parental homeownership. As Möhring (2012) has argued, this method forms a robust alternative to multilevel models in cases where the number of observations at the higher level is small (see also Dewilde and Stier 2014). We used a slightly different procedure for homeownership rates and the growth in these rates. Logically, the likelihood of becoming a homeowner would be associated with homeownership rates: The larger the stock of owner-occupied homes, the more likely a transition to homeownership. The intergenerational transmission of homeownership would be less strong in countries where the growth in homeownership is stronger: In such countries, there are fewer parents who can pass on homeownership. We therefore included the rate itself as a main effect and the growth as an interaction term with parental homeownership. A final model includes all macro-level indicators at the same time. The standard errors of the models were corrected for the clustering of observations in period-country combinations.^6^ We also estimated separate models per country; the results of these were largely in line with the results we present and can be obtained from the authors.

## Results

### Models Without Macro-Level Indicators

From Models 1 and 2 in Table 4, it can be seen that, before controlling for any other variables, a positive effect of parental homeownership on the likelihood of a transition to homeownership is found for most, but not all, countries. The main effect pertains to the reference country, the Netherlands, and is estimated at 0.214 (*p* < 0.01) for men and 0.189 (*p* < 0.01) for women. This corresponds to hazard ratios (exponentiated coefficients) of 1.24 and 1.21: According to the model, Dutch men (women) whose parents were homeowners were 1.24 (1.21) times as likely to become homeowners in a given year as those whose parents were not. For most countries, the effects were not significantly different (see interaction terms), but for France it was estimated to be smaller, for Danish and Belgian women it was larger and for Danish men it was smaller. For Swedish men, and Spanish men and women, no effect of parental homeownership was found: The positive main effect was offset by a negative interaction term that was just as large. These effects are more or less robust to the inclusion of variables indicating parental and individual characteristics as well as degree of urbanization (Models 3 and 4 in Table 4). It is noteworthy that the pattern of cross-national differences does not correspond to expectations derived from ideas about unitary versus dual rental markets or welfare regimes: The differences in the effects of parental homeownership are just as large within as between different types of rental markets (the Netherlands, Sweden, Denmark, Germany and Switzerland as unitary rental markets vs. France, Belgium, Italy, Spain and Greece as dual markets) and welfare regimes (the Netherlands, Sweden and Denmark as representatives of the social-democratic regime; Germany, Switzerland, France and Belgium as representatives of the conservative regime; Italy, Spain and Greece as representatives of the southern European regime).

**Table 4:** Logistic regression results using country-specific fixed effects

	(1)		(2)		(3)		(4)	
	Men		Women		Men		Women	
	Coef	SE	Coef	SE	Coef	SE	Coef	SE
Parents homeowner [age 10; main effect (Netherlands)]	0.21***	0.03	0.19***	0.02	0.27***	0.05	0.20***	0.05
Parents homeowner (age 10) * Sweden	−0.26**	0.12	0.01	0.06	−0.33*	0.18	−0.11**	0.05
Parents homeowner (age 10) * Denmark	−0.10*	0.06	0.14***	0.05	−0.01	0.07	0.15**	0.07
Parents homeowner (age 10) * Germany	−0.06	0.15	−0.04	0.09	−0.09	0.16	−0.11	0.11
Parents homeowner (age 10) * Switzerland	0.06	0.11	0.07	0.06	0.04	0.12	0.06	0.09
Parents homeowner (age 10) * France	−0.13***	0.05	−0.12***	0.05	−0.22***	0.06	−0.16**	0.07
Parents homeowner (age 10) * Belgium	0.04	0.07	0.13***	0.04	−0.10	0.11	−0.03	0.08
Parents homeowner (age 10) * Italy	−0.08	0.08	0.04	0.06	−0.09	0.11	−0.05	0.07
Parents homeowner (age 10) * Spain	−0.34***	0.07	−0.22***	0.05	−0.26***	0.07	−0.32***	0.08
Parents homeowner (age 10) * Greece	−0.06	0.17	0.13	0.17	0.02	0.21	0.17	0.19
Sweden	0.58**	0.27	0.41*	0.24	0.52**	0.21	0.34***	0.10
Denmark	0.73***	0.26	0.50*	0.27	0.70***	0.13	0.43***	0.09
Germany	−0.62***	0.20	−0.62***	0.21	−0.72***	0.11	−0.73***	0.11
Switzerland	−0.49**	0.20	−0.73***	0.22	−0.63***	0.21	−0.78***	0.17
France	0.20	0.24	0.07	0.23	0.14	0.13	0.06	0.13
Belgium	0.19	0.29	0.21	0.26	0.18	0.18	0.21	0.13
Italy	−0.19	0.22	−0.21	0.22	−0.06	0.12	0.01	0.07
Spain	0.26	0.22	0.16	0.26	0.51***	0.09	0.64***	0.06
Greece	0.08	0.25	−0.16	0.21	0.16	0.21	−0.01	0.19
Parent’s job (age 10; ref. = elementary)								
Less skilled job					−0.05	0.04	0.08**	0.04
Technician/ass. professional					0.03	0.08	0.20***	0.07
Professional					0.03	0.06	0.14***	0.05
Unknown/armed forces/no breadwinner					0.04	0.07	−0.07	0.07
Rural/village (age 10; ref.)								
Moderately urban					−0.04	0.04	−0.10***	0.03
Large city					−0.23***	0.04	−0.23***	0.05
Job level (ref. = elementary)								
Less skilled job					0.06	0.05	0.16***	0.04
Technician/ass. professional					0.23***	0.07	0.24***	0.08
Professional					0.30***	0.07	0.31***	0.07
Unknown/armed forces					−0.14	0.11	0.15	0.18
Never in paid work					−0.09	0.16	−0.02	0.06
Employed (ref.)								
Non-employed					−0.32**	0.16	0.00	0.07
In education					−1.17***	0.14	−0.62***	0.09
Years in labor market					−0.01*	0.01	0.01***	0.00
Education (in years)					0.04***	0.01	0.05***	0.01
Age					0.01	0.02	−0.02	0.01
Age^2^					0.00**	0.00	0.00**	0.00
Married (ref.)								
Never partnered					−2.68***	0.22	−2.77***	0.24
Cohabiting unmarried					−0.34***	0.12	−0.30***	0.10
Widowed/divorced					−0.70***	0.08	−0.74***	0.08
No children (ref.)								
1–2 children					−0.43***	0.11	−0.48***	0.12
3 or more children					−0.58***	0.12	−0.54***	0.11
Constant	−3.51***	0.18	−3.44***	0.17	−2.36***	0.36	−1.95***	0.28
Observed N person-years	179,327		202,349		179,327		202,349	
Observed calendar years	1965–2009		1965–2009		1965–2009		1965–2009	
Number of clusters	50		50		50		50	
Pseudo-*R* ^2^	0.01		0.02		0.11		0.11	
Model Chi-square	392		567		6321		16,859	

Robust standard errors are corrected for the clustering of respondents in country–period combinations. All individual-level variables are time-varying except those measured at respondent’s age 10

* *p* < 0.10; ** *p* < 0.05; *** *p* < 0.01

The main effects of the country dummies signify cross-national differences in the extent to which people become homeowners, and the speed of this process. The effects of the variables indicating parental and individual characteristics as well as the degree of urbanization were largely in line with previous research. Perhaps most noteworthy are various gender differences. Parental socioeconomic status, measured as occupation, was estimated to be more influential to women’s than to men’s homeownership (compare Blaauboer 2010), whereas men’s unemployment and enrollment in education had a stronger effect than women’s. Surprisingly, a negative effect of men’s work experience was found, likely because it was strongly associated with age as men spend long life-course spans in the labor market; for women, the age effect was smaller and the effect of work experience was in the expected positive direction.

### Findings for the Macro-Level Indicators

The effects of the macro-level indicators and their interactions with parental homeownership were all in the expected direction (Table 5). For women, all effects were statistically significant except those involving homeownership rates and the maximum loan-to-value ratio (both main effects and interactions with parental homeownership were not significant), and for men all were significant except the effects involving the maximum loan-to-value ratio. The higher the average house price in a country and period, relative to GDP per capita, and so the less affordable homeownership was, the smaller the likelihood of entry to homeownership in a year and the stronger the impact of parental homeownership. The higher the level of economic affluence (measured in GDP per capita) was, the greater the likelihood of a transition to homeownership and the smaller the effect of parental homeownership. Logically, people were more likely to become homeowners in a year in contexts with higher levels of homeownership. And, as expected, in countries with a strong growth of homeownership rates (as in some southern European countries), the association between parents’ and children’s homeownership was less strong, as the parental generation was logically less likely to be in homeownership. These effects, however, were only significant for men, not for women.

**Table 5:** Logistic regression results using country-specific fixed effects and macroeconomic variables

	(1)		(2)		(3)		(4)	
	Men		Men		Men		Men	
	Coef	SE	Coef	SE	Coef	SE	Coef	SE
Parents homeowner [age 10; main effect (Netherlands)]	0.01	0.06	0.41***	0.06	0.46*	0.28	0.38**	0.16
Parents homeowner (age 10) * Sweden	−0.25*	0.15	−0.31**	0.13	−0.29*	0.18	−0.45*	0.25
Parents homeowner (age 10) * Denmark	−0.04	0.11	−0.01	0.12	−0.01	0.07	0.01	0.13
Parents homeowner (age 10) * Germany	−0.31*	0.18	−0.08	0.14	−0.10	0.16	−0.01	0.22
Parents homeowner (age 10) * Switzerland	−0.23	0.16	0.15	0.13	0.01	0.15	−0.18	0.18
Parents homeowner (age 10) * France	−0.28***	0.06	−0.23***	0.06	−0.22***	0.05	−0.16	0.16
Parents homeowner (age 10) * Belgium	−0.17***	0.05	−0.12	0.09	−0.11	0.11	0.00	0.15
Parents homeowner (age 10) * Italy	−0.19***	0.04	−0.10	0.08	−0.14	0.12	0.00	0.11
Parents homeowner (age 10) * Spain	−0.37***	0.06	−0.32***	0.06	−0.28***	0.07	−0.11	0.14
Parents homeowner (age 10) * Greece	−0.46***	0.15	−0.05	0.19	−0.18	0.21	−0.18	0.24
Sweden	0.47***	0.18	0.53***	0.17	0.46**	0.20	0.31	0.25
Denmark	0.74***	0.12	0.71***	0.14	0.70***	0.13	0.41**	0.21
Germany	−0.51***	0.11	−0.72***	0.09	−0.72***	0.12	−0.81***	0.17
Switzerland	−0.39**	0.19	−0.76***	0.17	−0.51**	0.25	−0.49*	0.27
France	0.19***	0.07	0.14	0.09	0.12	0.13	−0.35**	0.17
Belgium	0.23**	0.12	0.19	0.15	0.19	0.18	−0.56	0.39
Italy	0.04	0.07	−0.04	0.08	0.04	0.15	−0.99***	0.26
Spain	0.62***	0.13	0.58***	0.09	0.52***	0.09	−0.81*	0.44
Greece	0.62***	0.21	0.25	0.19	0.37	0.26	−0.96**	0.44
House price-to-GDP ratio (per country and period)	−0.04***	0.01						
Parents homeowner (age 10) * house price-to-GDP ratio	0.04***	0.01						
GDP (× 1000 current USD per country and period)			0.01***	0.00				
Parents homeowner (age 10) * GDP (×1000)			−0.01***	0.00				
Maximum loan-to-value ratio (per country and period)					0.00	0.00		
Parents homeowner (age 10) * maximum loan-to-value ratio					0.00	0.00		
Homeownership rates (% per country and period)							0.03**	0.01
Parents homeowner (age 10) * homeownership growth							0.00*	0.00
Observed N person-years	177,553		177,553		166,936		84,752	
Observed calendar years	1965–2009		1965–2009		1965–2009		1965–2009	
Number of clusters	47		47		47		60	
Pseudo-*R* ^2^	0.11		0.11		0.11		0.14	
Model Chi-square	11,900		12,948		10,511		5417	

	(5)		(6)		(7)		(8)	
	Women		Women		Women		Women	
	Coef	SE	Coef	SE	Coef	SE	Coef	SE
Parents homeowner [age 10; main effect (Netherlands)]	0.00	0.05	0.31***	0.03	0.56**	0.22	0.06	0.10
Parents homeowner (age 10) * Sweden	−0.04	0.05	−0.08	0.07	−0.05	0.05	0.03	0.11
Parents homeowner (age 10) * Denmark	0.12***	0.04	0.15***	0.04	0.15**	0.06	0.25***	0.07
Parents homeowner (age 10) * Germany	−0.28***	0.10	−0.10	0.07	−0.11	0.10	0.07	0.14
Parents homeowner (age 10) * Switzerland	−0.18***	0.06	0.12**	0.06	−0.04	0.04	0.01	0.15
Parents homeowner (age 10) * France	−0.21***	0.05	−0.16***	0.04	−0.14**	0.06	0.06	0.08
Parents homeowner (age 10) * Belgium	−0.08**	0.04	−0.04	0.05	−0.03	0.07	0.05	0.07
Parents homeowner (age 10) * Italy	−0.14	0.09	−0.06	0.05	−0.15	0.10	0.03	0.05
Parents homeowner (age 10) * Spain	−0.42***	0.05	−0.37***	0.06	−0.33***	0.06	−0.15	0.10
Parents homeowner (age 10) * Greece	−0.20	0.15	0.12	0.18	−0.01	0.19	0.43**	0.21
Sweden	0.29***	0.09	0.33***	0.09	0.27***	0.10	0.12	0.19
Denmark	0.47***	0.07	0.44***	0.08	0.42***	0.08	0.31**	0.15
Germany	−0.56***	0.09	−0.74***	0.09	−0.73***	0.11	−0.89***	0.13
Switzerland	−0.56***	0.13	−0.86***	0.14	−0.60***	0.14	−0.75***	0.21
France	0.11	0.08	0.07	0.09	0.04	0.13	−0.27*	0.14
Belgium	0.26***	0.08	0.22**	0.10	0.22*	0.12	−0.08	0.26
Italy	0.10	0.11	0.04	0.06	0.12	0.10	−0.34	0.25
Spain	0.74***	0.11	0.70***	0.08	0.65***	0.07	0.16	0.48
Greece	0.35**	0.18	0.05	0.17	0.16	0.21	−0.50	0.37
House price-to-GDP ratio (per country and period)	−0.03***	0.01						
Parents homeowner (age 10) * house price-to-GDP ratio	0.03***	0.01						
GDP (×1000 current USD per country and period)			0.01***	0.00				
Parents homeowner (age 10) * GDP (×1000)			−0.01***	0.00				
Maximum loan-to-value ratio (per country and period)					0.00	0.00		
Parents homeowner (age 10) * maximum loan-to-value ratio					0.00	0.00		
Homeownership rates (% per country and period)							0.01	0.01
Parents homeowner (age 10) * homeownership growth							0.00	0.00
Observed N person-years	199,999		199,999		189,348		99,133	
Observed calendar years	1965–2009		1965–2009		1965–2009		1965–2009	
Number of clusters	47		47		47		60	
Pseudo-*R* ^2^	0.11		0.11		0.11		0.12	
Model Chi-square	71,104		82,777		59,208		5753	

Robust standard errors are corrected for the clustering of respondents in country–period combinations. Extra variables that are controlled for: parent’s job at age 10, degree of urbanization, current or last job, employment status, years in labor market, education, age, marital status and number of children. These results can be obtained from the authors. The number of observations is lower for these specifications—compared to Table 4—because we only included those individuals for whom the macro-level variables are available. For most specifications, three clusters are missing, which are Denmark, Sweden and Switzerland for the period after 2000. For specifications (4) and (8), individuals are only included if ‘homeownership rate at age 10’ was known. We need this variable to calculate the homeownership growth rate

* *p* < 0.10; ** *p* < 0.05; *** *p* < 0.01

The correlations between the macro-level indicators were not extremely strong (Table 6), and therefore, we were confident enough to estimate models in which all macro-level indicators were included at the same time (Table 7). The only effects that remained statistically significant in these models were those of the interactions between parental homeownership and the affordability indicator. For men, there is an unexpected positive interaction between parental homeownership and the maximum loan-to-value ratio. Perhaps this is related to the fact that house prices tend to be higher (and homeownership hence less affordable) in contexts with fewer mortgage restrictions. All other macro-level effects became smaller than in the separate models and some changed sign.

**Table 6:** Correlations between macro-indicators

	Price-to-GDP ratio	Per capita GDP	Maximum loan-to-value	Homeownership rate	Homeownership growth	Price-to-income ratio
Price-to-GDP ratio	1					
Per capita GDP	0.13	1				
Maximum loan-to-value	0.16	−0.09	1			
Homeownership rate	−0.29	−0.41	−0.04	1		
Homeownership growth	−0.28	0.10	0.33	0.06	1	
Price-to-income ratio^a^	0.46	0.27	−0.18	−0.38	−0.03	1

^a^Price-to-income ratio has only been used in sensitivity analysis

**Table 7:** Alternative specification including all macroeconomic variables

	(1)		(2)	
	Men		Women	
	Coef	SE	Coef	SE
Parents homeowner [age 10; main effect (Netherlands)]	−0.77**	0.33	−0.39	0.33
Parents homeowner (age 10) * Sweden	−0.48**	0.20	0.09	0.15
Parents homeowner (age 10) * Denmark	0.02	0.15	0.23***	0.09
Parents homeowner (age 10) * Germany	−0.22	0.26	−0.10	0.12
Parents homeowner (age 10) * Switzerland	−0.28	0.30	−0.25	0.30
Parents homeowner (age 10) * France	−0.20	0.18	0.01	0.11
Parents homeowner (age 10) * Belgium	0.01	0.13	0.02	0.11
Parents homeowner (age 10) * Italy	0.15	0.15	0.00	0.12
Parents homeowner (age 10) * Spain	−0.20*	0.11	−0.21	0.15
Parents homeowner (age 10) * Greece	−0.29	0.30	0.16	0.26
Sweden	0.38	0.27	0.18	0.21
Denmark	0.50*	0.29	0.47**	0.19
Germany	−0.69***	0.22	−0.90***	0.16
Switzerland	−0.38	0.42	−0.86**	0.34
France	−0.27	0.17	−0.18	0.13
Belgium	−0.43	0.49	0.20	0.31
Italy	−0.92	0.56	0.08	0.40
Spain	−0.58	0.75	0.70	0.62
Greece	−0.69	0.96	0.14	0.61
House price-to-GDP ratio (per country and period)	−0.04*	0.02	−0.01	0.02
Parents homeowner (age 10) * House price-to-GDP ratio	0.06***	0.02	0.04**	0.02
GDP (×1000 current USD per country and period)	0.00	0.01	0.01	0.01
Parents homeowner (age 10) * GDP (×1000)	0.00	0.01	0.00	0.01
Maximum loan-to-value ratio (per country and period)	0.00	0.01	0.00	0.01
Parents homeowner (age 10) * maximum loan-to-value ratio	0.01**	0.00	0.00	0.00
Homeownership rates (% per country and period)	0.02	0.02	−0.01	0.02
Parents homeowner (age 10) * homeownership growth	0.00	0.00	0.00	0.00
Parent’s job (age 10; ref. = elementary)				
Less skilled job	0.00	0.05	0.09*	0.05
Technician/ass. professional	0.03	0.10	0.23***	0.08
Professional	0.09	0.08	0.09	0.06
Unknown/armed forces/no breadwinner	0.10	0.09	−0.07	0.11
Rural/village (age 10; ref.)				
Moderately urban	−0.02	0.05	−0.09**	0.05
Large city	−0.24***	0.07	−0.22***	0.06
Job level (ref. = elementary)				
Less skilled job	0.17**	0.07	0.12**	0.05
Technician/ass. professional	0.33***	0.09	0.15*	0.09
Professional	0.38***	0.10	0.32***	0.08
Unknown/armed forces	−0.07	0.19	0.29	0.24
Never in paid work	−0.15	0.20	−0.07	0.09
Employed (ref.)				
Non-employed	−0.34*	0.19	−0.02	0.09
In education	−1.14***	0.20	−0.62***	0.09
Years in labor market	0.01	0.01	0.02***	0.00
Education (in years)	0.04***	0.01	0.05***	0.01
Age	0.10**	0.04	0.03	0.04
Age^2^	0.00***	0.00	0.00**	0.00
Married (ref.)				
Never partnered	−2.69***	0.23	−2.90***	0.26
Cohabiting unmarried	−0.49***	0.12	−0.39***	0.10
Widowed/divorced	−0.89***	0.12	−0.86***	0.10
No children (ref.)				
1–2 children	−0.45***	0.13	−0.45***	0.14
3 or more children	−0.75***	0.13	−0.52***	0.14
Constant	−4.21***	0.82	−2.56***	0.79
Observed N person-years	78,961		92,948	
Observed calendar years	1965–2009		1965–2009	
Pseudo-*R* ^2^	0.13		0.11	

Robust standard errors are corrected for the clustering of respondents in country–period combinations. Individuals are only included if ‘homeownership rate at age 10’ was known (compare Models 4 and 8 in Table 5)

* *p* < 0.10; ** *p* < 0.05; *** *p* < 0.01

As one would expect, the inclusion of macro-level variables led to changes in the main effect of parental homeownership and the interactions with the country dummies, and so, in the estimated differences between countries in the impact of parental homeownership. But these differences did not become smaller, and neither did they become more in line with expected differences between unitary and dual rental markets and among welfare regimes. The differences looked just as erratic as they did before introducing the macro-level indicators.

## Discussion

Parental homeownership is known to be positively associated with the likelihood of adult children of becoming first-time homeowners. We investigated to what extent this association differs between ten countries in Europe, using SHARELIFE-data. We tested hypotheses on the impact of contextual differences in housing affordability, economic affluence, access to mortgages and levels of homeownership as well as growth in these levels on the likelihood of a transition to first-time homeownership in a given year—that is, the occurrence and timing of such a transition, using a relatively new method proposed by Möhring (2012) and used previously by Dewilde and Stier (2014): Logistic regression of person-years including fixed effects for countries and interactions between parental home-ownership and macro-level indicators measured for country-period combinations. We also assessed to what extent differences in the impact of parental homeownership were in line with differences between unitary and dual rental markets, or with differences among social-democratic, conservative and southern European welfare regimes (countries representing liberal market regimes were not in our data).

Several hypotheses on the differences we could test using macro-level indicators were supported: Intergenerational transmission of homeownership was stronger in contexts where house prices were higher and thus homeownership was less affordable, and it was less strong in more affluent contexts and in contexts where homeownership had grown more (men only). However, remaining differences in the effect of parental homeownership across countries could not in any way be attributed to type of rental market (unitary or dual) or welfare regime. Apparently, the remaining differences in the intergenerational transmission of homeownership are caused by other factors than the ‘usual suspects’ of comparisons across European countries: Differences we could not investigate. Such differences could be related to the tax treatment of gifts and inheritances, patterns of geographical mobility and immobility, cultural differences in homeownership or parental assistance not covered by differences among welfare regimes, policy differences, or idiosyncratic differences in habits around homeownership that we do not observe.

Of the macro-level factors associated with the intergenerational transmission of homeownership, affordability stood out as the most important: It was the only determinant that remained statistically significant after all other indicators were accounted for. While it is often assumed that housing market-related changes have resulted in increased dependence on parental resources, our paper provides more formal evidence of the existence of an effect of housing market characteristics in a comparative context. This finding is important because it indicates that adult children rely more on their parents in contexts where homeownership is less affordable, regardless of the underlying forces responsible for affordability issues. In such contexts, it is all the more likely that inequalities in homeownership and housing wealth are exacerbated as a result of the intergenerational transmission of homeownership (compare Kurz and Blossfeld 2004a, b). Our findings are also relevant for current debates on (un)affordable housing and on the tightening of mortgage regulations for young people following the economic crisis (e.g., Ronald and Elsinga 2011)—both imply a higher dependence of children on parental wealth with regard to the transition to homeownership and its associated social and economic benefits.

Possibly, future research using different data could solve part of the remaining puzzle. Including countries representing liberal welfare regimes could be one of the ways to go, although it will be a challenge to harmonize the SHARE data with similar surveys from other countries such as the English Longitudinal Study of Ageing (ELSA).

Some strengths of our analyses were the use of internationally harmonized data and the inclusion of macro-level indicators in the analyses of the intergenerational transmission of homeownership. Among the weaknesses was the limited measurement of homeownership and the transition to it. As in many other datasets [with the notable exception of the Netherlands Kinship Panel Study (NKPS)], homeownership was treated as an attribute of a dwelling (‘was this house owned’) rather than a combination of persons and a dwelling (‘who owned this house’). And as in all datasets we know of, transitions to homeownership could only be detected if they coincided with a move. While this is true of most transitions to homeownership in most countries, this excludes a sizeable number of transitions in others. Another weakness concerns the use of parental homeownership as a proxy for parental assistance, as we could only control partly for other known mechanisms responsible for the intergenerational transmission of homeownership, such as socioeconomic status transmission. The fact that several of our hypotheses—which were derived from the idea that the ‘need’ for parental help varies across countries and contexts—were confirmed, strengthens confidence in our findings. Finally, our choice to use macro-level indicators that refer to country-period combinations implies that we do not explicitly focus on trends over time in the association between parents’ and children’s homeownership. Empirical evidence from other studies suggests that this association has strengthened over time. We do, however, find clear evidence that housing market-related determinants—in particular affordability—matter; these determinants are often, but casually, mentioned as ‘culprits’ in the literature. Future research could disentangle the concept of ‘affordability’ by focusing on the drivers of its components, i.e., income and housing cost.

In all, we believe that our findings make an interesting contribution to the literature on the transition to homeownership, the intergenerational transmission of homeownership and contextual differences in this transmission.

## Footnotes

1 Another mechanism is inheritance, but this is less relevant for younger people entering first-time homeownership.

2 With the exception of Greece, which is the only country where homeownership rates were already high in 1960.

3 Note that this implies that in our event history analysis, we use left-censored data for some of the respondents. This is no problem because we know for all time units and respondents whether the transition to homeownership has taken place and we also know the duration from the start of the spell.

4 For most countries, homeownership rates before the 1980s (and for many countries also after the 1980s) are only available for certain years. We collected data for 1970, 1980, 1990, 2000, 2004 and 2008. These rates were then also used for the adjacent years. For example, we used the figure for 1970 for the period 1965–1975. The Figure 2008 was used for the period 2007–2010.

5 Because the data pertain to main respondents and their partners, the observations for male and female respondents are not independent of each other. It is therefore not appropriate to perform statistical tests on the differences between men and women.

6 One could also think of a correction for the clustering of person-years in respondents. However, as Yamaguchi (1991) has shown, logistic regression models of person-years provide correct standard errors under normal conditions, making corrections unnecessary. An important condition is that the number of 1s in the dependent variable should not exceed 10 %, which is usually the case and also in our data: In most calendar years, it is below 5 %, and in some it is around 6 %.
